# 25-Hydroxycholesterol restricts human norovirus replication in human intestinal enteroids

**DOI:** 10.1128/jvi.01109-25

**Published:** 2025-10-21

**Authors:** Verónica Costantini, Wadzanai P. Mboko, Helen Wall, Kimberly Huynh, Anna M. Montmayeur, Geun Woo Park, Ruijie Xu, Jan Vinjé, Preeti Chhabra

**Affiliations:** 1 Viral Gastroenteritis Branch, Division of Viral Diseases, National Center for Immunization and Respiratory Diseases, Centers for Disease Control and Prevention 209773, Atlanta, Georgia, USA; 2 Cherokee Nation Operational Solutions, Tulsa, Oklahoma, USA; Loyola University Chicago - Health Sciences Campus, Maywood, Illinois, USA

**Keywords:** human intestinal enteroids, 25-hydroxycholestrol, norovirus

## Abstract

Norovirus is the leading cause of acute gastroenteritis worldwide. Since the development of human intestinal enteroid (HIE) cell culture systems, significant advancements have been made in exploring and evaluating therapeutic strategies to combat human norovirus infections. Here, we investigate whether 25-hydroxycholesterol (25-HC), a key regulator of cholesterol homeostasis and bile acid production, can restrict human norovirus replication in jejunal HIE monolayers. Jejunal HIE monolayers derived from two donors (J2 and J3 cell lines) were treated with increasing concentrations of 25-HC (ranging from 0.0001 µM to 5 µM) for 24 hours. Monolayers were then infected with norovirus GII.1[P33] or GII.4 Sydney[P31] and incubated for 24 hours at 37°C and 5% CO_2_. Viral RNA was quantified by reverse transcription-quantitative PCR at 1 hour post-infection (hpi) and 24 hpi. Regardless of HIE cell line or norovirus genotype, a dose-dependent restriction of human norovirus replication was observed after 25-HC treatment (0.07–1.23 log decrease). The top upregulated pathways associated with the 25-HC treatment included innate immunity and pro-inflammatory pathways, while the top downregulated pathways included cholesterol biosynthesis pathways. The 25-HC resulted in upregulation of genes associated with innate immune response, including C-C motif chemokine 2 (CCL2), CCL20, intercellular adhesion molecule 1 (ICAM1), and interleukin 23A (IL23A), and downregulation of genes related to cholesterol biosynthesis, including proprotein convertase subtilisin/kexin type 9 and acetyl-CoA acetyltransferase (ACAT2). Our data suggest that modulating the downstream signaling pathway of 25-HC has therapeutic potential as a strategy to control human norovirus infections and enhance antiviral immune responses.

**IMPORTANCE:**

Human noroviruses are a leading cause of both epidemic and endemic acute gastroenteritis worldwide. Currently, there are no licensed vaccines or antiviral treatments available. The immune response to human norovirus infection remains incompletely understood. The development of the human intestinal enteroid (HIE) system has revolutionized norovirus research by enabling detailed investigations into viral replication, evaluation of potential control strategies, and analysis of host cellular responses involving both innate and adaptive immunity. In recent years, 25-hydroxycholesterol (25-HC) has emerged as a key regulator of cellular metabolism and antiviral defense. In this study, we investigated the antiviral effects of 25-HC on human norovirus using the HIE culture system. Our findings highlight the therapeutic potential of 25-HC in controlling human norovirus infections and enhancing antiviral immune responses, thereby contributing to a deeper understanding of host restriction mechanisms in viral infections.

## INTRODUCTION

Human noroviruses are a leading cause of both epidemic and endemic acute gastroenteritis worldwide, with an estimated attributable death of 35,914 globally, primarily in young children in developing countries (1). Norovirus illness is characterized by the sudden onset of diarrhea and/or vomiting, often accompanied by nausea, abdominal pain, fever, headache, and body aches (2). Symptoms typically appear 12 to 48 hours after exposure and are generally self-limiting, resolving within 1 to 3 days. Noroviruses belong to the family *Caliciviridae* and are single-stranded, positive-sense RNA viruses. Phylogenetically, they are classified into 10 genogroups (GI–GX), with genogroups GI and GII accounting for the majority of human infections (3). Since 2002, GII.4 strains have been responsible for at least half of all norovirus outbreaks and cases of acute gastroenteritis worldwide (4, 5).

Currently, there are no licensed vaccines or antiviral treatments for norovirus; however, several vaccine candidates are in various stages of development (6). The immune response to human norovirus infection remains incompletely understood. However, the development of the human intestinal enteroid (HIE) system has provided new insights into host factors that influence virus replication (7–9). HIEs are primary cells derived from intestinal biopsies, cultured in a three-dimensional format, and later seeded as monolayers to support human norovirus replication (7, 10). This innovative culture system has revolutionized norovirus research, enabling detailed investigation of viral replication, evaluation of control strategies, and analysis of host cellular responses related to innate and adaptive immunity (11, 12).

In recent years, oxysterols have emerged as key regulators of both innate and adaptive immunity (13). Among them, 25-hydroxycholesterol (25-HC) is well established as a critical mediator of cholesterol homeostasis (14–16) and bile acid synthesis (17). More recently, 25-HC has also been recognized for its broad-spectrum antiviral activity. It exerts its antiviral effects through multiple mechanisms, including altering the positioning, orientation, and solvent accessibility of membrane cholesterol. These changes compromise the stability and integrity of cholesterol-rich membranes, hindering viral fusion with the host cell membrane and thereby blocking viral entry (18). In addition, 25-HC can directly inhibit viral replication (19). It also promotes the release of inflammatory cytokines and modulates immune responses, thereby contributing to antiviral defense (20).

Several studies have demonstrated that 25-HC restricts the replication of both enveloped and non-enveloped viruses (20–25). Notably, pretreatment of RAW 264.7 cells with 25-HC significantly reduced murine norovirus replication (26). However, the impact of 25-HC on human norovirus replication has not yet been explored. In this study, we investigated the antiviral effects of 25-HC on human norovirus using the HIE cell culture system.

## RESULTS

**Fig 1:**
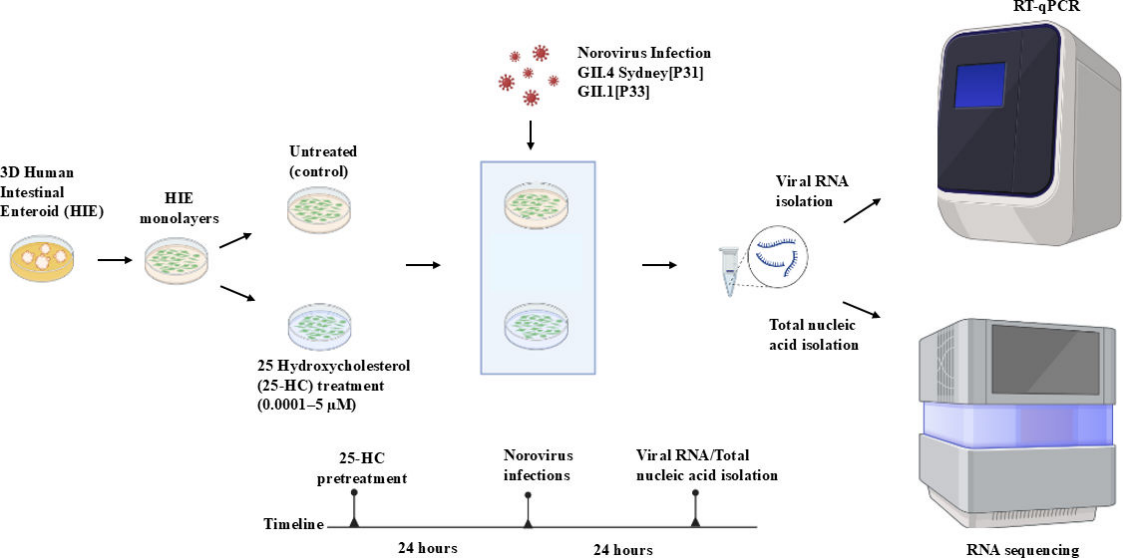
Experimental design. HIE monolayers derived from J2 and J3 lines were generated from 3D cultures and differentiated for 4 days. Differentiated monolayers were treated for 24 hours with 25-HC in differentiation medium (treated), or with differentiation medium alone (control). Following pretreatment, cells were infected with human norovirus strains GII.4 Sydney[P31] or GII.1[P33]. Viral RNA levels were quantified by RT-qPCR, and host gene expression profiles were analyzed by bulk RNA sequencing.

**Fig 2:**
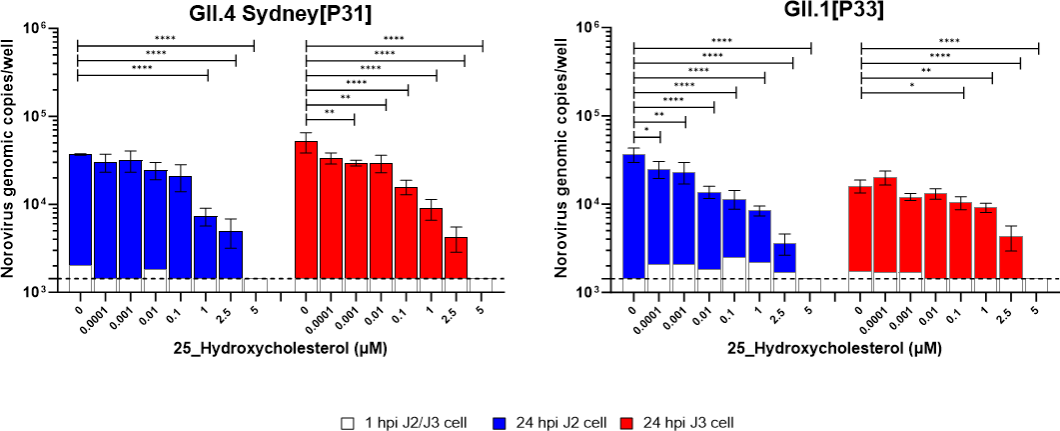
25-HC treatment significantly reduces human norovirus replication in HIE monolayers. Jejunal HIE monolayers derived from two donors (J2 and J3 cell lines) were treated with increasing concentrations of 25-HC (0.0001–5 µM). After 24 hours of treatment, monolayers were infected with human norovirus strains GII.4 Sydney[P31] or GII.1[P33] and incubated for 24 hours at 37°C and 5% CO₂. Viral RNA levels were quantified by RT-qPCR at 1 hpi and 24 hpi. Data are presented as mean ± SD from two independent experiments, each with three technical replicates. *P*-values were determined using one-way ANOVA followed by Sidak’s multiple comparisons test (*P ≤  0.1, **P  ≤ 0.01, ***P  ≤  0.001, ****P  ≤  0.0001).

To assess whether 25-HC restricts human norovirus replication in HIE monolayers, we performed a dose-response experiment with norovirus GII.4 Sydney[P31] in a J2 HIE cell line pretreated with increasing concentrations of 25-HC (0.0001–5 µM) (Fig. 1 and 2). In the absence of 25-HC, GII.4 Sydney[P31] showed a 1.2-log increase in genomic copies at 24 hours post-infection (hpi). Treatment with 1–5 µM 25-HC significantly reduced viral replication, resulting in a 0.6–1.2-log decrease in genomic copies at 24 hours compared to HIEs not treated with 25-HC (*P* < 0.0001). Given the donor-specific origin of HIE lines, we next examined whether 25-HC could similarly restrict GII.4 Sydney[P31] replication in the J3 HIE line. In the absence of 25-HC, GII.4 Sydney[P31] showed a 1.5-log increase in genomic copies at 24 hpi. In comparison, treatment with 0.001–0.01 µM 25-HC reduced the increase in genomic copies by 0.3-log (*P* =  0.006), while 0.1–5 µM resulted in a 0.5–1.5-log reduction (*P* < 0.0001) (Fig. 2).

**Fig 3:**
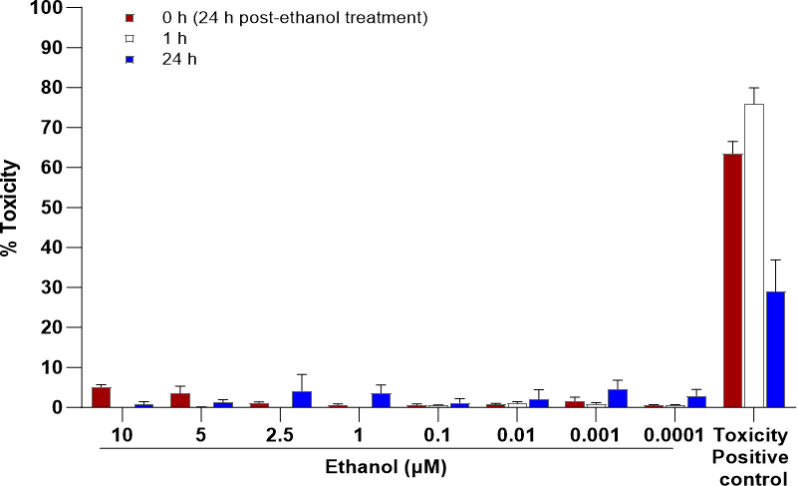
Ethanol cytotoxicity. Jejunal HIE monolayers derived from two donors (J2 and J3) were treated with increasing concentrations of ethanol (0.0001–10 µM) and incubated for 24 hours at 37°C. Cytotoxicity was assessed at 0, 1, and 24 hours post-treatment. Data are presented as mean ± SD from three technical replicates.

To determine whether these findings extend to other norovirus genotypes, we tested 25-HC against GII.1[P33] in both J2 and J3 cell lines. Without 25-HC, GII.1[P33] showed a 1.4-log increase in J2 and approximately a 1-log increase in J3. Similar to GII.4 Sydney[P31], 25-HC significantly inhibited GII.1[P33] replication in J2, with concentrations of 0.01–5 µM resulting in a 0.6–1.2-log decrease compared to untreated controls (*P* < 0.0001). In J3, treatment with 0.1 µM 25-HC decreased viral replication by 0.1-log (*P*  =  0.0128), while 2.5 and 5 µM treatments led to 0.6- and 0.9-log reductions, respectively (*P* < 0.0001) (Fig. 2). Since ethanol was used as the solvent for 25-HC, a dose-dependent cytotoxicity assessment of ethanol (0.0001–10 µM) was performed on HIE monolayers. No cytotoxic effects were observed (Fig. 3). Overall, these results indicate that 25-HC inhibits replication of human noroviruses GII.4 Sydney[P31] and GII.1[P33] in a dose-dependent manner across both jejunal HIE lines. While sensitivity to 25-HC treatment varied by virus strain and cell line, treatment with ≥ 2.5 µM 25-HC consistently resulted in a significant reduction in viral replication (0.8–1.5-log decrease; *P* < 0.0001).

**Fig 4:**
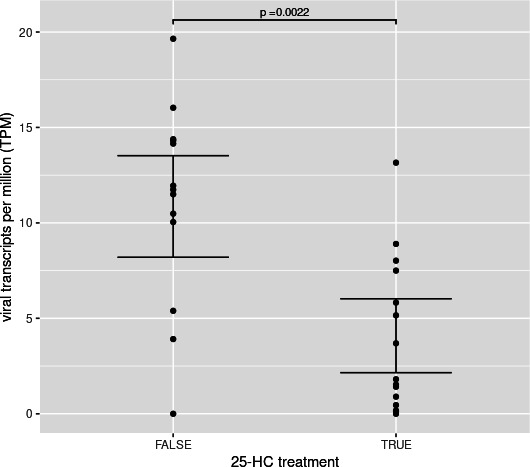
25-HC reduces norovirus transcript abundance in infected HIE monolayers. RNA transcripts from norovirus-infected J2 and J3 HIE monolayers were aligned to the GII.4 Sydney[P31] and GII.1[P33] reference genomes. All virus-exposed samples, regardless of cell line or viral genotype, were included in the analysis. Viral transcript abundance was significantly reduced in 25-HC–treated samples. No significant differences in viral read counts were observed between J2 and J3 cell lines or between human norovirus strains GII.4 Sydney[P31] and GII.1[P33]. Statistical significance was assessed using the Mann-Whitney *U* test.

To gain insight into the mechanisms by which the cellular response to 25-HC restricts human norovirus replication, mRNA sequencing was performed on HIE monolayers (J2 and J3) pretreated with 5 µM 25-HC and subsequently infected with GII.4 Sydney[P31] and GII.1[P33] noroviruses (Fig. 1). A reduction in abundance of transcripts mapping to norovirus genomes in both cell lines confirmed that pretreatment with 5 µM 25-HC inhibited human norovirus replication (Fig. 4; Fig. S1). No significant differences in viral transcript levels were observed between the J2 and J3 cell lines or between the two viral genotypes.

**Fig 5:**
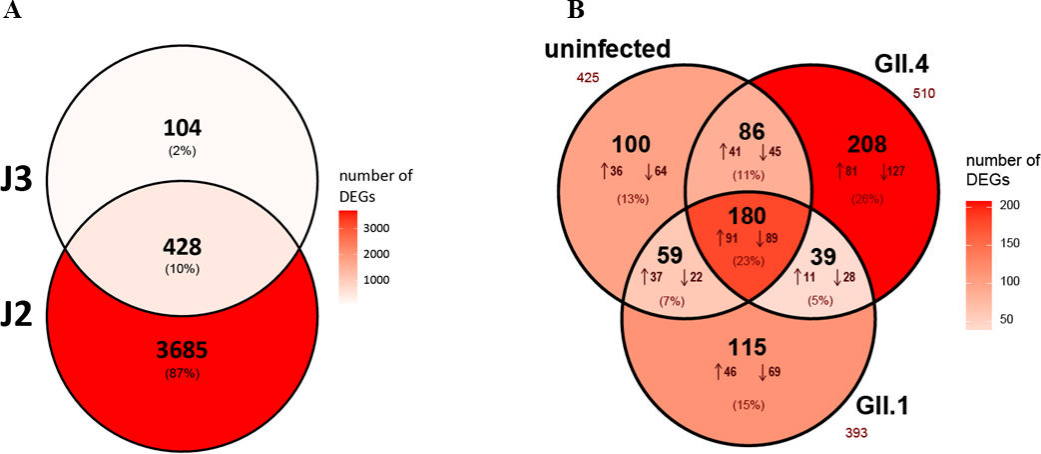
DE genes in HIE monolayers following 25-HC treatment. (**A**) Venn diagram showing the overlap of DE genes (absolute log₂ fold change >0.5, adjusted *P*-value < 0.05) in uninfected J2 and J3 HIE monolayers. (**B**) Overlap of DE genes shared between uninfected, GII.4 Sydney-infected, and GII.1-infected HIE monolayers in both J2 and J3 cell lines. Only genes with consistent directionality of expression change (up- or downregulated) across both cell lines were included to minimize donor-specific effects and highlight conserved responses to 25-HC treatment.

**Fig 6:**
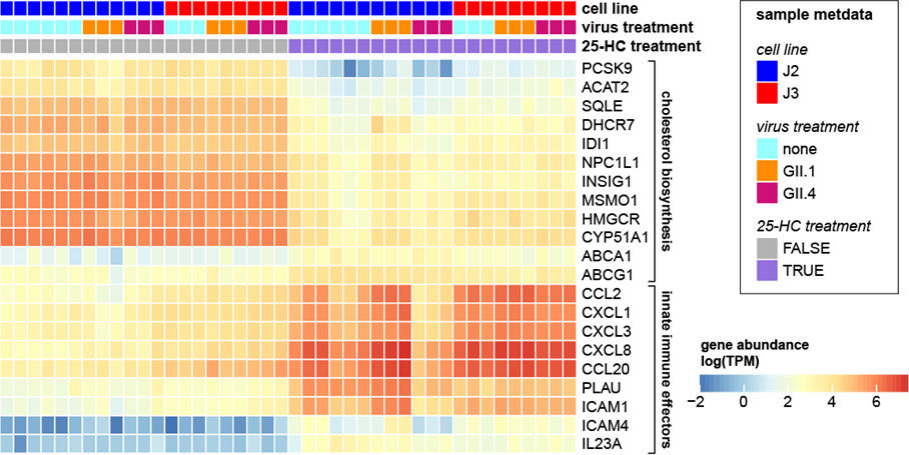
Heatmap of DE genes in 25-HC-treated HIE monolayers compared to untreated controls. Genes included in the heatmap were selected from cholesterol biosynthesis and innate immune effector pathways. Gene expression is represented as log-transformed transcripts per million (TPM). Differential expressions were determined by RNA sequencing analysis.

Differential gene expression analysis revealed a substantially higher number of differentially expressed (DE) genes in response to 25-HC in uninfected J2 HIE monolayers (*n* = 4,113) compared to J3 (*n* = 532) (Fig. 5A). A total of 180 DE genes were commonly regulated across all viral treatments and HIE cell lines following 25-HC treatment (supplementary material), comprising 91 upregulated and 89 downregulated genes (Fig. 5B). Among the most significantly upregulated genes shared across all conditions were several key innate immune effectors, including intercellular adhesion molecule 1 (ICAM1), plasminogen activator urokinase (PLAU), and pro-inflammatory cytokines such as CCL2, CXCL1, CXCL3, CXCL8, and CCL20 (Fig. 6).

Conversely, the most significantly downregulated genes were predominantly involved in cholesterol biosynthesis. These included proprotein convertase subtilisin/kexin type 9 (PCSK9), acetyl-CoA acetyltransferase 2 (ACAT2), 3-hydroxy-3-methylglutaryl-CoA reductase (HMGCR), 7-dehydrocholesterol reductase (DHCR7), methylsterol monooxygenase 1 (MSMO1), squalene epoxidase (SQLE), and sterol 14α-demethylase (CYP51A1) (Fig. 6). These findings suggest that 25-HC modulates both innate immune responses and key enzymatic pathways involved in cholesterol biosynthesis.

**Fig 7:**
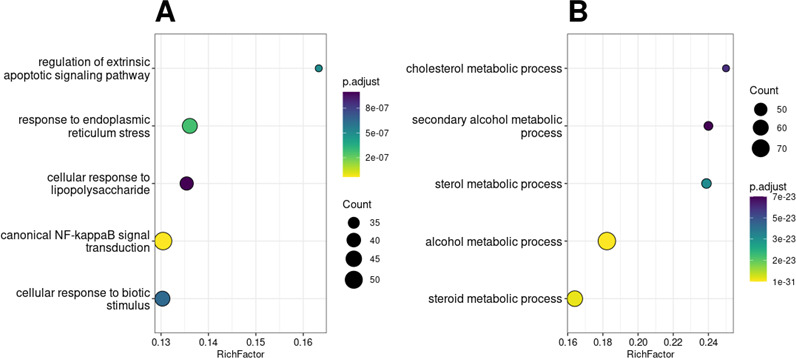
Pathway over-representation analysis of 25-HC-treated HIE monolayers. Top 10 upregulated (**A**) and downregulated (**B**) GO pathways identified in 25-HC-treated HIE monolayers compared to untreated controls. Pathways are ranked by adjusted *P*-values, indicated by dot color. Dot size represents the number of DE genes associated with each pathway. The *x*-axis (RichFactor) denotes the ratio of DE genes to the total number of genes in the respective pathway. DE genes used in this analysis are found in the supplementary material.

To gain a broader understanding of the cellular pathways affected by 25-HC, gene set over-representation analysis of Gene Ontology (GO) biological processes was performed on the up- and downregulated DE genes. This analysis identified 815 upregulated and 386 downregulated pathways. The top enriched pathways represented by upregulated genes were associated with innate immunity and pro-inflammatory responses, including canonical NF-κB signaling and regulation of the extrinsic apoptotic signaling pathway (Fig. 7A). In contrast, pathways significantly enriched among downregulated genes were primarily related to cholesterol biosynthesis and metabolic processes (Fig. 7B). Genes DE in J2 but not in J3 were involved in pathways closely related to those of the shared genes—such as stress response, fatty acid metabolism, and organic acid biosynthesis (Fig. S2)—suggesting that J2 HIEs exhibit a similar but more amplified transcriptional response.

## DISCUSSION

We report that 25-HC exhibits antiviral activity against human norovirus in HIEs. Pretreatment of HIEs with 25-HC prior to infection significantly inhibited viral replication in a dose-dependent manner. Additionally, 25-HC treatment induced upregulation of innate immune response pathways and downregulation of cholesterol biosynthesis in HIE cells.

The development of HIE cell culture system for human norovirus replication has opened new avenues for investigating viral inactivation, innate immune responses, antiviral susceptibility, neutralizing antibody activity, and other key aspects of norovirus biology (7, 8, 11, 27–31). Norovirus replication in HIE exhibits both HIE line-specific and strain-specific differences (31). Since each HIE line represents a unique individual donor, we employed two distinct HIE lines (J2 and J3) and two norovirus genotypes (GII.1[P33] and GII.4 Sydney[P31]) to ensure robustness and reproducibility of our findings. The antiviral effects of 25-HC were consistently observed across both HIE lines and against both GII genotypes, supporting its broad-spectrum antiviral activity as previously reported for other viruses, including rotavirus and murine norovirus (23–26, 32–34). Notably, one HIE line (J2) showed a greater number of DE genes in response to 25-HC treatment; the overall pathways affected by these genes were similar across both lines. Such variation in cellular responses between HIE lines is expected due to differences in donor genomic backgrounds and life histories (35).

25-HC plays multifaceted roles in the antiviral response by significantly modulating cholesterol homeostasis, inflammation, and innate immune signaling (36). The interplay between cholesterol regulatory pathways and inflammatory signaling is critical to the development, progression, and resolution of infectious diseases (37). As a key regulator of cholesterol homeostasis, 25-HC modulates intracellular cholesterol levels by influencing the expression of enzymes involved in cholesterol biosynthesis, as well as transport proteins responsible for cholesterol uptake and efflux (38). It is well established that 25-HC effectively suppresses the transcription of genes involved in cholesterol synthesis in cultured cells (39, 40). We found that treatment of HIE monolayers with 25-HC led to downregulation of cholesterol biosynthesis and transport pathways. Notably, the expression of key genes involved in cholesterol metabolism including PCSK9, ACAT2, SQLE, HMGCR, DHCR7, MSMO1, and CYP51A1 were significantly reduced, consistent with observations from previous studies in other cell culture models (39–41). Among these, PCSK9 and ACAT2 have emerged as promising targets for the development of new cholesterol-lowering therapies.

Statins are known to increase PCSK9 (critical regulator of cholesterol metabolism) activity and shown to enhance norovirus replication both in replicon-bearing cells and in gnotobiotic pigs (42–45). In this study, a reduction in norovirus replication in HIE cells was accompanied by decreased PCSK9 expression. Further investigation is needed to determine whether the use of PCSK9 inhibitors may also contribute to reducing norovirus susceptibility, especially in the elderly population. Similarly, a previous study reported that inhibition of ACAT2 significantly reduced norovirus replication in Huh-7 cells harboring a norovirus replicon (43). ACAT2 expression is restricted to hepatocytes and enterocytes, where it plays a key role in the esterification of free cholesterol during intestinal cholesterol absorption (46). Given that enterocytes are the primary site of norovirus replication (7, 47), our observed association between decreased ACAT2 expression and reduced viral replication warrants further investigation.

In addition to regulating cholesterol biosynthesis during viral infections, 25-HC has emerged as an important regulator of inflammatory and innate immune responses. It is a metabolic product of cholesterol, generated through the conversion of cholesterol by cholesterol-25-hydroxylase, an enzyme encoded by the interferon-stimulated gene CH25H (25, 48). Under normal conditions, CH25H is expressed at low levels in human cells; however, its expression is significantly upregulated in response to interferons, particularly during viral infections (49). This upregulation leads to increased production of 25-HC, which enhances immune cell activation and promotes the synthesis of various immune mediators (37, 49). Our data demonstrate that treatment of HIE monolayers with 25-HC induced a significant upregulation of pro-inflammatory genes—such as CCL2, CCL20, CXCL1, CXCL3, and CXCL8—as well as immune response genes including ICAM1, ICAM4, PLAU, and interleukin (IL) 23A. Chemokines with C-X-C and C-C motifs, particularly CCL2, are commonly expressed during various viral infections (50). More recently, elevated levels of CCL20 have been reported in rotavirus infections (51). IL23 is known to regulate intestinal microbial homeostasis and plays a role in mediating immunopathological responses during murine *Campylobacter jejuni* infection (52, 53). The role of IL23 and its derivatives in human norovirus infection remains to be fully explored. These findings indicate that 25-HC may confer protection to intestinal epithelial cells by priming innate immune defenses prior to viral challenge (24).

Our study has several limitations. First, experiments were conducted using only two HIE lines, and additional HIE lines should be evaluated to better represent the broader human population. Second, the virus inocula were derived from original stool samples, meaning that other stool components may have influenced the observed gene expression results. To overcome these potential differences, we focused our analysis on findings that were consistent across both HIE cell lines and norovirus genotypes. While certain genes associated with cholesterol metabolism and innate immunity were DE, validating their role in susceptibility to norovirus infection requires more in-depth analysis. This aspect was not addressed, as it was beyond the scope of the current study.

In summary, 25-HC has emerged as a key regulator of both cellular metabolism and antiviral defense. Its presence can induce a broad antiviral state, providing more extensive protection compared to many other interferon-stimulating gene-mediated effects. Overall, our study highlights the therapeutic potential of 25-HC in controlling human norovirus infection. A deeper understanding of host restriction strategies in viral infections holds significant promise for uncovering novel mechanisms and developing innovative antiviral approaches.

## MATERIALS AND METHODS

### HIE cell culture

Secretor-positive jejunal 3D HIE cultures (J2 and J3 lines) were maintained as previously described (8). After 7 days, 3D cultured organoids were dissociated into single-cell suspensions and re-suspended in IntestiCult Human Organoid Growth Medium (OGM; STEMCELL Technologies) supplemented with 10 mM Y-27632 (Sigma-Aldrich). Cells were then plated as undifferentiated monolayers in collagen IV-coated 96-well plates (Sigma-Aldrich). After 24 hours, differentiation was initiated by replacing the medium with differentiation media, and the cultures were incubated for 4 days at 37°C. The differentiation medium consisted of Advanced DMEM/F12 supplemented with 1% GlutaMAX, 1% penicillin/streptomycin, 1% 1 M HEPES, 1:100 B27 supplement, 1:50 N2 supplement, 1:1,000 mouse epidermal growth factor (all from Invitrogen), 1 mM N-acetyl-L-cysteine, 10 nM [Leu15]-gastrin, 10 µM Y-27632 (all from Sigma-Aldrich), 500 nM A83-01 (Tocris), and 20% noggin-conditioned medium (homemade) (Fig. 1).

### Ethanol cytotoxicity

As ethanol was used as the solvent for 25-HC, its cytotoxic effects on HIE monolayers were evaluated. Differentiated monolayers were treated with ethanol concentrations ranging from 0.0001 to 10 µM and incubated for 24 hours at 37°C. Cytotoxicity was assessed using the CyQUANT LDH Cytotoxicity Assay Kit (Thermo Fisher Scientific, Waltham, MA, USA), following the manufacturer’s protocol. Differentiated HIE monolayers (J2 or J3 cell lines) were cultured in 96-well plates in triplicate. To establish baseline and maximum lactate dehydrogenase (LDH) release, wells were treated with infection media (baseline control) and lysis buffer (positive control), respectively. Experimental wells were treated with ethanol at concentrations ranging from 0.0001 to 10 µM. After 0, 1, and 24 hours, the supernatants were transferred to a new plate and mixed with detection reagent. Plates were incubated at room temperature in the dark for 30 minutes, after which stop solution was added. Absorbance was measured at 490 nm, with background correction at 680 nm, using a BioTek Epoch 2 microplate reader. Cytotoxicity was calculated based on the corrected absorbance values.

### Oxysterol pretreatment of HIE

Serial dilutions of 25-HC were prepared in differentiation media supplemented with 500 µM sodium glycochenodeoxycholate (GCDCA; Sigma-Aldrich) and 50 µM ceramide (C2; Santa Cruz Biotechnology), referred to as GCDCA/C2. Differentiated HIE monolayers (J2 and J3 cell lines) were treated with either (i) increasing concentrations of 25-HC (0.0001–5 µM) or (ii) GCDCA/C2-supplemented differentiation media alone (untreated control). Cells were incubated at 37°C for 24 hours (Fig. 1).

### Infection and viral RNA detection

Duplicate monolayers (treated and untreated, J2 and J3 lines) were inoculated with norovirus strains GII.4 Sydney[P31] (5.85 × 10⁴ copies/well) and GII.1[P33] (4.76 × 10⁴ copies/well) using 10% stool filtrates diluted in infection media. Both norovirus-positive stool samples were pre-screened for 14 pathogens that are included in xTAG Gastrointestinal Pathogen Panel and were positive only for noroviruses (data not shown). Following a 1-hour incubation at 37°C with 5% CO₂, monolayers were washed twice with CMGF- (advanced DMEM/F12 supplemented with 1% GlutaMAX, 1% penicillin/streptomycin, and 1% 1 M HEPES). Subsequently, 100 µL of differentiation media supplemented with GCDCA/C2 was added to each well. For each infection set, one plate was immediately frozen at −70°C to serve as a baseline, while a duplicate plate was incubated at 37°C with 5% CO₂ for 24 hours before freezing. Infection media consisted of CMGF- supplemented with GCDCA/C2 (54). Cell lysates and supernatants were collected at 1 and 24 hpi. Viral RNA was extracted and quantified by reverse transcription-quantitative PCR (RT-qPCR) as described (4, 55) (Fig. 1). Data represent means ± standard deviation (SD) from *n* = 2 independent experiments, each with three technical replicates. Statistical analysis of infectivity assays was performed using one-way analysis of variance (ANOVA) followed by Holm-Sidak’s multiple comparisons test (*P ≤ 0.1, **P ≤ 0.01, ***P ≤ 0.001, ****P ≤ 0.0001). All analyses were conducted using GraphPad Prism 10.0 (GraphPad Software, La Jolla, CA, USA).

### Detection of cellular gene expression

RNA was extracted from cell lysates of the following groups using the MagMAX−96 Total RNA Isolation Kit (Thermo Fisher Scientific): (i) uninfected J2 and J3 cell lines treated with 25-HC (5 µM); (ii) J2 and J3 cell lines treated with 25-HC and infected with either GII.4 Sydney[P31] or GII.1[P33] viruses; (iii) untreated J2 and J3 cell lines infected with GII.4 Sydney[P31] or GII.1[P33] viruses; and (iv) untreated, uninfected J2 and J3 cell lines. RNA samples were subjected to bulk RNA sequencing using 2 × 150 bp paired-end reads on the Illumina NovaSeq platform (Fig. 1).

### RNA sequence data processing

Raw sequencing reads were quality-checked using FastQC (https://www.bioinformatics.babraham.ac.uk/projects/fastqc/). Reads were filtered with fastp to remove those with a Phred quality score below Q15 in more than 40% of bases and those exhibiting low complexity (<30%) (56). Adapter sequences were also trimmed using fastp. Cellular gene expression was quantified using salmon quant with options --gcBias and --seqBias (57), employing the human reference index and annotation from Gencode v44 (58). To detect viral RNA, a chimeric human-viral genome was created by concatenating the human reference genome (GRCh38, release 110) (59) with the sequences for norovirus strains GII.4 Sydney[P31] (OL913976) and GII.1[P33] (OL898506). Reads were aligned to this chimeric genome and quantified using STAR v2.7.11 with option --quantMode GeneCounts (60). Statistical significance of differences in viral transcript abundance between pairwise conditions was assessed using the Mann-Whitney *U* test via the wilcox.test function in R.

### Differential gene expression

DE genes between experimental conditions were identified using the DESeq method implemented in the R package DESeq2, applying the default Wald test on salmon abundance estimates. Adjusted *P*-values were calculated using the Benjamini-Hochberg procedure to control for false discovery rate (61). Genes were considered DE if they had an absolute log₂ fold-change greater than 0.5 and an adjusted *P*-value below 0.05. Venn diagrams illustrating gene overlaps were generated using the R package ggVennDiagram, while heatmaps displaying log-transformed transcript abundance (transcripts per million) were created with the R package pheatmap.

### Pathway over-representation analysis

GO biological processes enriched or suppressed were identified by performing over-representation analysis on lists of up- and down-regulated DE genes using the enrichGO function from the R package clusterProfiler (62). This analysis included all DE genes identified from the comparison of 25-HC-treated enteroids versus untreated controls, regardless of cell line or viral infection status. Over-representation *P*-values were adjusted using the Benjamini-Hochberg method (61) and GO terms with adjusted *P* < 0.05 were considered statistically significant. Top pathways were selected based on Rich Factor (ratio of DE genes in the process to the total genes in the process).

## Data Availability

The sequences generated from RNA sequencing are uploaded to NCBI SRA under BioProject PRJNA1295912 (accession numbers SRR34772610 to SRR34772651).

## SUPPLEMENTAL MATERIAL

Figure S1 jvi.01109-25-s0001.tif Abundance of viral transcripts in RNA-seq data across sample groups.

Figure S2 jvi.01109-25-s0002.tif Pathway over-representation analysis of uninfected and 25-HC-treated (A) J2 and (B) J3 HIE monolayers.

Supplemental tables jvi.01109-25-s0003.xlsx Differential gene expression.

ASM does not own the copyrights to Supplemental Material that may be linked to, or accessed through, an article. The authors have granted ASM a non-exclusive, world-wide license to publish the Supplemental Material files. Please contact the corresponding author directly for reuse.
