# Supplementary figures and images for: 25-Hydroxycholesterol restricts human norovirus replication in human intestinal enteroids
Source: J Virol. 2025 Oct 21;99(11):e01109-25. doi: 10.1128/jvi.01109-25 (PMC12645967; doi:10.1128/jvi.01109-25)

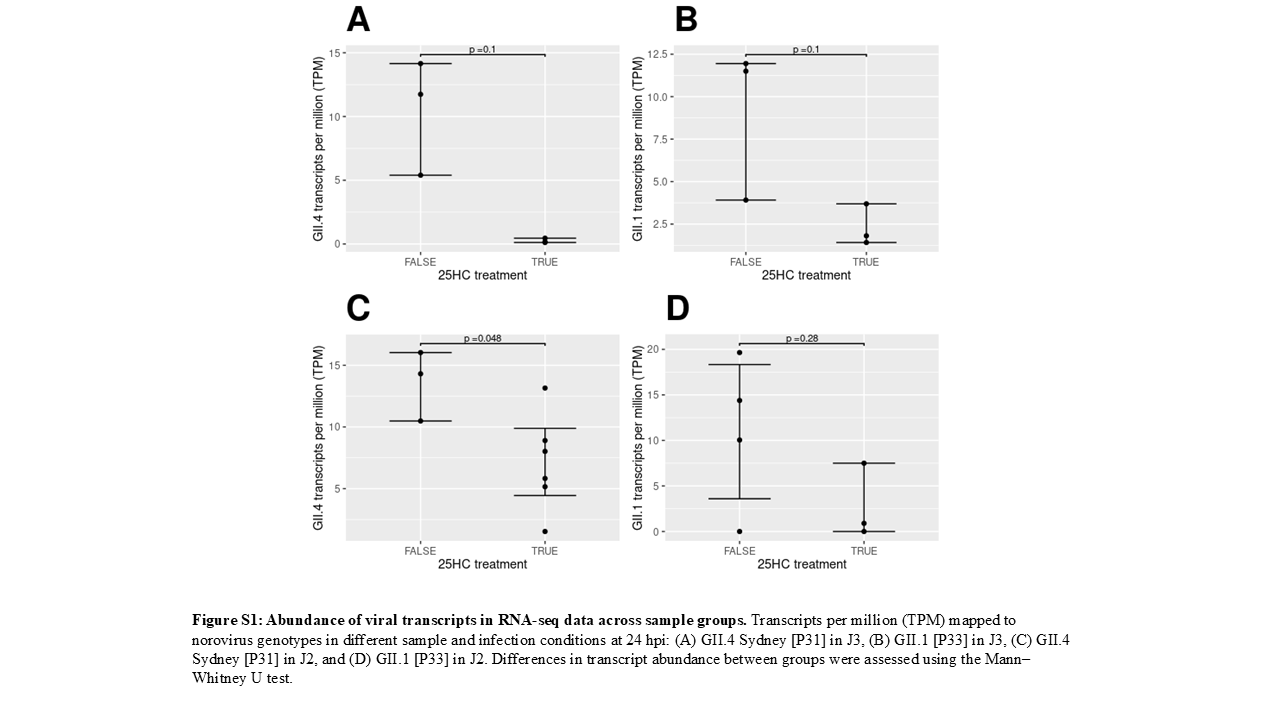

Supplement: Figure S1 — Abundance of viral transcripts in RNA-seq data across sample groups. [file jvi.01109-25-s0001.tif]

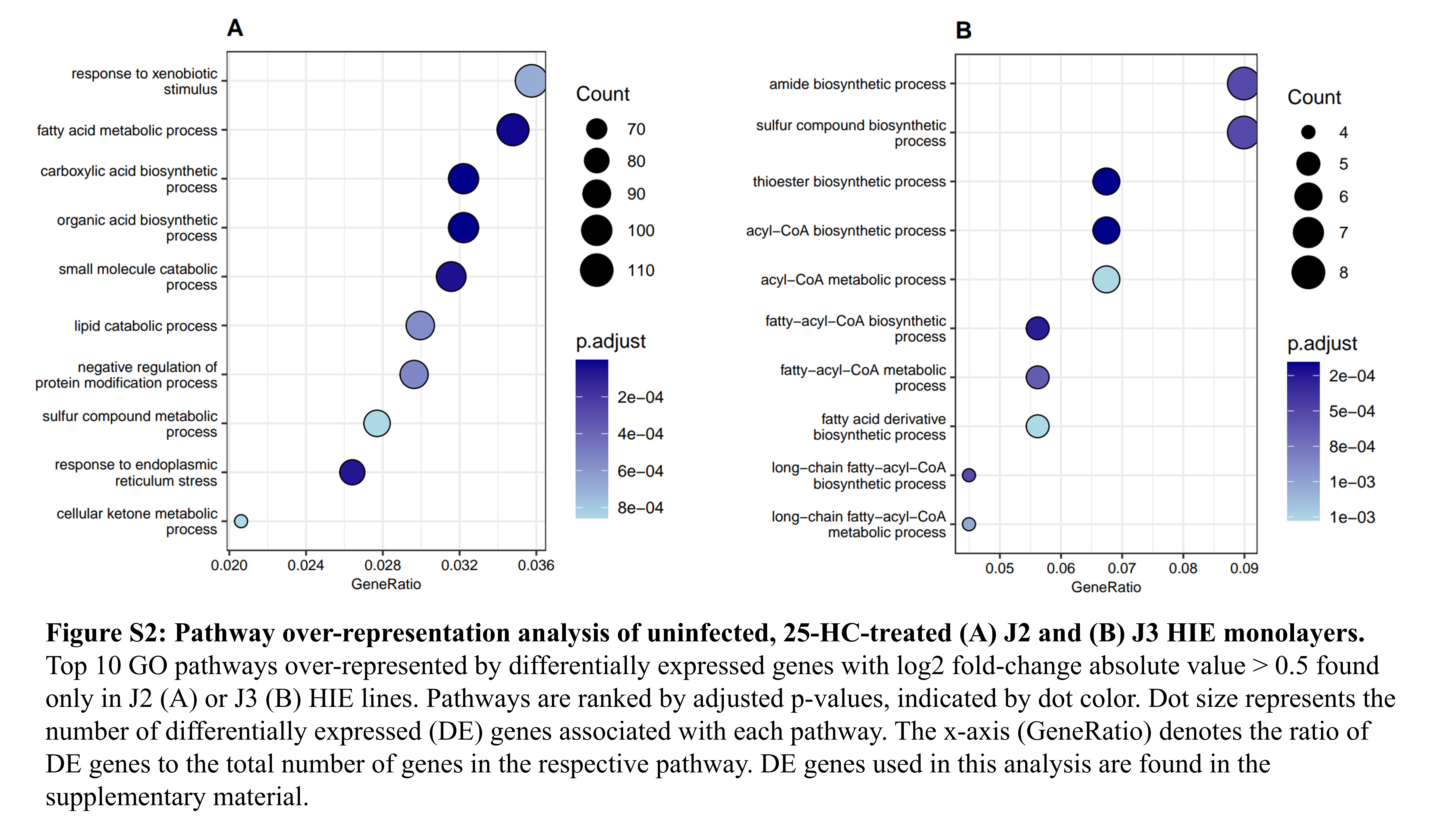

Supplement: Figure S2 — Pathway over-representation analysis of uninfected and 25-HC-treated (A) J2 and (B) J3 HIE monolayers. [file jvi.01109-25-s0002.tif]
